# Differential Expression of PDGF Receptor-α in Human Placental Trophoblasts Leads to Different Entry Pathways by Human Cytomegalovirus Strains

**DOI:** 10.1038/s41598-020-57471-3

**Published:** 2020-01-23

**Authors:** Zin Naing, Stuart T. Hamilton, Wendy J. van Zuylen, Gillian M. Scott, William D. Rawlinson

**Affiliations:** 1 Serology and Virology Division, Department of Microbiology, NSW Health Pathology, Prince of Wales Hospital, Sydney, New South Wales Australia; 2 School of Women’s and Children’s Health, Faculty of Medicine, University of New South Wales, Sydney, New South Wales Australia; 3 School of Medical Sciences, Faculty of Medicine, University of New South Wales, Sydney, New South Wales Australia; 4 School of Biotechnology and Biomolecular Sciences, University of New South Wales, Sydney, New South Wales Australia

**Keywords:** Viral infection, Herpes virus, Viral transmission

## Abstract

Human cytomegalovirus (CMV) is the leading non-genetic cause of fetal malformation in developed countries. CMV placental infection is a pre-requisite for materno-fetal transmission of virus, and fetal infection. We investigated the roles of the viral pentameric complex gH/gL/pUL128-pUL131A, and cellular platelet-derived growth factor receptor-α (PDGFRα) for CMV infection in first trimester extravillous-derived (SGHPL-4) and villous-derived (HTR-8/SVneo) trophoblast cells. Infection with four CMV clinical and laboratory strains (Merlin, TB40E, Towne, AD169), and Merlin deletion mutants of UL128-, UL130-, and UL131A-genes, showed a cell type-dependent requirement of the viral pentameric complex for infection of trophoblast cells. The viral pentameric complex was essential for infection of villous trophoblasts, but non-essential for extravillous trophoblasts. Blocking of PDGFRα in extravillous trophoblasts, which naturally express PDGFRα, inhibited entry of pentameric complex-deficient CMV strains, but not the entry of pentameric positive CMV strains. Transient expression of PDGFRα in villous trophoblasts, which are naturally deficient in PDGFRα, promoted the entry of CMV strains lacking gH/gL/pUL128-pUL131A, but had no effect on entry of pentameric positive CMV strains. These results suggest PDGFRα is an important cell receptor for entry of CMV mutant strains lacking gH/gL/pUL128-pUL131A complexes in some placental cells, suggesting these entry pathways could be potential antiviral targets.

## Introduction

Human cytomegalovirus (CMV) is the leading viral cause of congenital infection in developed countries, with a global incidence of 0.3–0.7% of all live births^1,2^. Congenital CMV infection may result in fetal death^3,4^ or permanent neurodevelopmental sequelae including sensorineural hearing loss and mental disability^5^. The risk of congenital CMV is highest in pregnant women who acquire primary CMV during pregnancy, with 32% chance of transmission to the fetus^1,6^. Reactivation of latent virus or re-infection with a new strain of CMV can also lead to congenital CMV transmission, but at a much lower transmission rate of 1.4%^1^. CMV transmission can occur throughout pregnancy, although congenital infection during the first trimester of pregnancy presents the greatest risk to the developing fetus in terms of disease severity and long-term sequelae^3,7^.

The exact mechanism by which CMV transmission occurs *in utero* is unknown. However, evidence suggests CMV infection of the placenta is a critical step in materno-fetal transmission of the virus^8,9^. CMV infection of placental trophoblasts has been demonstrated in placental tissue from congenitally infected infants using immunohistochemical and *in situ* PCR techniques^10,11^. CMV-DNA, CMV-transcripts and CMV-proteins from immediate-early (IE), early (E), and late times have been detected in all placental cell types of naturally-infected, early and late gestation placentae^12,13^. Productive CMV replication has also been demonstrated in *ex vivo* villous explant models, where CMV infection of villous cytotrophoblasts precedes infection of other placental cell types^14,15^. These observations indicate trophoblast cells are important sites for placental CMV infection and most likely transmission of the virus to the fetus.

In addition to placental explant models, studies have utilised primary cytotrophoblasts^16^, syncytiotrophoblast-like cells^16–19^, and trophoblast-derived cell lines^19–21^ to investigate CMV infection *in vitro*. Laboratory strains of CMV such as AD169 and Towne have been used most frequently for *in vitro* studies^16–22^. These CMV strains are known to have significant alterations in their genomes due to extensive propagation in cell culture^23–25^. The AD169, Towne and other CMV strains passaged extensively in fibroblasts have frame-shift mutations in one of three genes (UL128, UL130 and UL131A) of the UL128 locus^24,26^. Intact UL128 protein (pUL128), pUL130, and pUL131A can independently assemble onto glycoprotein H (gH)/gL heterodimers to form the gH/gL/pUL128-131A pentameric complex that is critical for entry into epithelial cells, endothelial cells, monocytes, and dendritic cells^27–31^. Mutations within the UL128 locus inhibit formation of the pentameric complex in AD169 and Towne strains and render these viruses incapable of infecting epithelial cells, endothelial cells, monocytes and dendritic cells.

The CMV gH/gL proteins also assemble into complexes with gO to form gH/gL/gO trimers, which is a necessary pre-requisite for virus entry into fibroblasts^30,32–35^. Some studies show gO promotes the incorporation of gH/gL into the virion, and gH/gL, but not gH/gL/gO, mediates CMV entry into fibroblasts^36,37^. However, further investigation from the same group has revealed all strains of CMV contain different compositions of gH/gL/gO trimers and gH/gL/pUL128-131A pentamers, and these envelope complexes are necessary for virus entry into host cells^38^. Interference studies through overexpression of gH/gL/pUL128-131A or gH/gL/gO in susceptible cells suggest these glycoprotein complexes bind to cell type-specific receptors, leading to resistance to CMV entry^35,39^. In addition to these envelope glycoprotein complexes, CMV gB homodimers are also essential for CMV entry into host cells via fusion with the plasma membrane^40–42^. A recent study has demonstrated gB, but not gH/gL/pUL128-pUL131A complex is required for CMV infection of placental trophoblast progenitor cells^43^. Since the human placenta is comprised of diverse trophoblast populations, including trophoblast progenitor cells, syncytiotrophoblasts, cell column cytotrophoblasts (proximal and distal), and extravillous trophoblasts, investigating the significance of viral pentameric complex on infection of these trophoblast cell types is of critical importance, with such studies of relevance for pathogenesis and vaccine research^44^.

The platelet-derived growth factor receptor-α (PDGFRα), epidermal growth factor receptor (EGFR), integrins, annexin II, dendritic cell-specific ICAM-grabbing nonintegrin, neurophilin-2 and OR14I1 have been proposed as CMV receptors for infection of different cell types including fibroblasts, epithelial cells, endothelial cells, lymphocytes, and dendritic cells^45–53^. Several of these receptors (PDGFRα, EGFR, integrin β1/αVβ3, annexin II) interact with gB, gH, or gO and this has been shown to facilitate viral entry and activation of cellular signalling pathways^45,46,48,54–56^. In placental trophoblasts, co-expression of EGFR and integrins (αVβ3 and α1β1) has been associated with increased susceptibility to CMV infection and viral replication^57^. Although the roles of PDGFRα in CMV cell entry have been inconsistent between studies of different cell types and virus strains, there is evidence for PDGFRα promoting entry of AD169 and UL131A-deleted CMV TR strains into epithelial and endothelial cells^58^. This suggests PDGFRα may be necessary for cellular entry of CMV strains lacking the pentameric gH/gL/pUL128-131A complex. Recent studies have shown virion gH/gL/gO binds to PDGFRα on the surface of fibroblasts and either directly or indirectly recruits gB to this complex to facilitate virus cell^59,60^ entry, with the N terminus of gO contributing to interactions with PDGFRα^56^.

Platelet derived growth factor (PDGF) consists of a family of homodimeric and heterodimeric forms of A- and B-chains and is generally described as a mitogen for fibroblasts, smooth muscle cells, and other cell types^61^. The PDGF ligands and their receptors, PDGFRα and PDGFRβ, are also expressed in different subpopulations of term placental extravillous trophoblasts, and reported to function as a growth factor in the development of cytotrophoblasts^62^. Therefore, we hypothesise that PDGFRα may be differentially expressed in diverse trophoblast cell types, and potentially influence the cellular entry of CMV into these cell types. In this research, we investigated the importance of viral gH/gL/pUL128-pUL131A complexes and cellular PDGFRα in CMV infection of two distinct trophoblast cell lines, extravillous-derived SGHPL-4 and villous derived HTR-8/SVneo cells. We demonstrated that CMV uses different entry pathways, involving at least the viral pentameric complex and cellular PDGFRα receptor for entry into placental trophoblasts.

## Materials and Methods

### Cells

Simian virus 40-transformed first trimester primary extravillous-derived trophoblasts (SGHPL-4 cells) were kindly provided by Guy Whitley (St. George’s University of London, Cranmer Terrace, London)^63^. These cells were cultured in F10 Ham’s medium (Life Technologies) containing 10% Fetal Bovine Serum (FBS) and 1% Penicillin Streptomycin Glutamine (PSG). Simian virus 40-transformed first trimester primary villous-derived trophoblasts (HTR-8/SVneo) were kindly provided by Charles Graham (The Department of Anatomy and Cell Biology, Queen’s University, Kingston) and Amanda Highet (School of Paediatrics and Reproductive Health, University of Adelaide)^64^. HTR-8/SVneo cells were maintained in RPMI-1640 Medium (Life Technologies) supplemented with 10% FBS and PSG. Cells were characterised by expression of trophoblast markers cytokeratin, human chorionic gonadotrophin, human placental lactogen, as well as *in vitro* invasiveness^63–65^. Retinal pigment epithelial (RPE-1) cells were kindly provided by Barry Slobedman (University of Sydney) and cultured in DMEM/F12 + GlutaMAX Medium (Life Technologies) containing 10% FBS and PSG. MRC-5 fibroblasts (ECACC, UK) were maintained in Modified Minimum Essential Medium (Life Technologies) containing 10% FBS and PSG.

### Viruses

A bacterial artificial chromosome (BAC) recombinant of CMV clinical strain Merlin, clone pAL1120 containing wild-type UL128 locus was kindly provided by Richard Stanton (Cardiff University)^66^. For reconstitution of Merlin, Merlin-BAC DNA was extracted from bacterial culture using Nucleobond BAC 100 extraction kit (Macherey-Nagel), then transfected into MRC-5 fibroblasts using Lipofectamine 2000 (Life Technologies). The reconstituted Merlin was then further propagated in RPE-1 epithelial cells to minimise mutation.

The UL128-, UL130-, and UL131A-deletion mutants of Merlin (Merlin ∆UL128, Merlin ∆UL130, Merlin ∆UL131A) were constructed by recombination in *Escherichia coli* SW102^67^. Briefly, primers with 3′ homology to the LacZ/Ampicillin/sacB cassette (~20 bp) and 5′ homology to sequences flanking the target viral gene (~80 bp) were used to amplify pAL1141 DNA^67^ and generate DNA fragments containing selectable markers with short regions of CMV homology. The sequences of primers used are listed in Table 1. Methylated template DNA was removed from PCR reactions by DpnI digestion (New England Biolabs), followed by gel purification using Wizard gel purification system (Promega). Purified PCR amplicons were electroporated into *E. coli* SW102 and colonies containing BAC with selectable markers were selected using ampicillin, X-gal and IPTG. In the second round of recombination, oligonucleotides with 50 bp homology to the left and right of the deleted gene were electroporated into *E. coli* SW102 resulting from the first round of recombination. Colonies containing correct oligonucleotide insertions were selected on media containing sucrose, X-gal and IPTG. Deletion of the target gene was confirmed by restriction digestion analysis and DNA sequencing of the gene region.

**Table 1:** Primers used for the construction of UL128-UL131A deletion mutants of Merlin and PDGFRα expression plasmids.

CMV/plasmid mutant	Primer	Primer sequence (5′–3′)
Merlin ΔUL128	UL128del-F (R1)	TCACTGCAGCATATAGCCCATTTTAGCGCGGCACACATCCAGCCGTTTGTGTTTCTTAACGCTCTCCAGGTACTGATCcctgtgacggaagatcacttcg
	UL128del-R (R1)	ATGAGTCCCAAAGATCTGACGCCGTTCTTGACGGCGTTGTGGCTGCTATTGGGTCACAGCCGCGTGCCGCGGGTGCGCGctgaggttcttatggctcttg
	UL128del-2 (R2)	**GCGACAGAAATCTCGAAACGCGTATTTCGGACAAACACACATTTTATTAT***GACGCGCGGTTTTCAAAATTCCCTGCGCGCGCGACGGGCTCAAACGATGA*
Merlin ΔUL130	UL130del-F (R1)	AGCCACAACGCCGTCAAGAACGGCGTCAGATCTTTGGGACTCATGACGCGCGGTTTTCAAAATTCCCTGCGCGCGCGAcctgtgacggaagatcacttcg
	UL130del-R (R1)	ATGCTGCGGCTTCTGCTTCGTCACCACTTTCACTGCCTGCTTCTGTGCGCGGTTTGGGCAACGCCCTGTCTGGCGTCTCctgaggttcttatggctcttg
	UL130del-2 (R2)	**TTGGGACTCATGACGCGCGGTTTTCAAAATTCCCTGCGCGCGCGACGGGC***ATTATTTCCCGTGACGCAGGCTAGTTGGCAAAGAGCCGCACGCTGAACTC*
Merlin ΔUL131A	UL131Adel-F (R1)	CTAGTTGGCAAAGAGCCGCACGCTGAACTCGAGGCTCCGGGCGTGTGGCGCCAGCGAACCGGCGGCGTTGAACGTGGTcctgtgacggaagatcacttcg
	UL131Adel-R (R1)	ATGCGGCTGTGTCGGGTGTGGCTGTCTGTTTGTCTGTGCGCCGTGGTGCTGGGTCAGTGCCAGCGGGAAACCGCGGAAActgaggttcttatggctcttg
	UL131Adel-2 (R2)	**AAAGTGGTGACGAAGCAGAAGCCGCAGCATATTATTTCCCGTGACGCAGG***GTTGCAGACTGAGAAAGAAAGCTTTATTATGAGACATCATACACATAGTA*
PDGFRα cloning	PDGFRA-Start	TAGTGGATCCATGGGGACTTCCCATCCGGCGTTC
	PDGFRA-Stop	CCGCTCGAGTTACAGGAAGCTGTCTTCCACCAGGTC
PDGFRα ΔD2-3	PDGFRA-2.3-F	ATACCTGCTGCC*C*G*C*CAGGCTACC
	PDGFRA-2.3-R	GGCAGCAGGTAT*A*A*T*GGCAGAATC
PDGFRα ΔD4-5	PDGFRA-4.5-F	ATAAGGAAAGAT*A*T*T*AAGAAATGT
	PDGFRA-4.5-R	ATCTTTCCTTAT*T*T*C*CTGAATCTT

Uppercase = CMV/PDGFRα homology; lower case = homology to template plasmid pAL1141; bold = homology to left of deleted gene; italic = homology to right of deleted gene; underlined = BamHI/XhoI restriction site; R1 = first round recombination; R2 = second round recombination; *phosphorothioate bond.

CMV TB40E-BAC4 was provided by Christian Sinzger (University Hospital Ulm, Germany) and reconstituted as described above. Merlin ∆UL128, Merlin ∆UL130, Merlin ∆UL131A, and TB40E were further propagated in MRC-5 fibroblasts for two to four passages following reconstitution. CMV laboratory strains, AD169 (ATCC, USA) and Towne (provided by Barry Slobedman, University of Sydney), were also propagated in MRC-5 cells. Cell-free supernatant of virus cultures were stored at −80 °C and standard plaque assay in MRC-5 cells was performed to measure the virus titre (pfu/cell) of all virus strains.

### PDGFRα expression vectors

Expression plasmids containing complete coding regions of human PDGFRα receptor were constructed by cDNA synthesis and PCR amplification of PDGFRα-specific sequences from RNA extracts of MRC-5 fibroblasts, followed by cloning into pcDNA3.1/V5-HisB plasmid (provided by Caroline Ford, University of New South Wales). Briefly, total RNA was extracted from MRC-5 cells using the RNAqueous kit (Ambion), and then cDNA was synthesized using iScript cDNA synthesis kit (BioRad), and PCR-amplified using primers containing PDGFRα-specific and restriction-site specific sequences (Table 1). PCR amplification was followed by restriction digestion (BamH1 and Xho1 restriction enzymes; New England Biolabs) and ligation of the DNA encoding PDGFRα receptor into pcDNA3.1/V5-HisB. The vector containing PDGFRα-specific cDNA sequence was transformed in *E. coli* JM109 (Promega), and a colony containing the desired insert was subjected to overnight culture in LB medium, followed by plasmid DNA extraction using PureLink HiPure plasmid midiprep kit (Life Technologies). In addition, commercially available expression plasmid pCMV-SPORT6 containing human PDGFRα cDNA (accession number: BC063414) was also obtained (Thermo Scientific).

The PDGFRα expression plasmids with deleted coding regions for extracellular domains 2–3 or 4–5, were constructed using the phosphorothioate method for the generation of deletion mutants from a plasmid DNA, as described previously^68^. The sequences of partially phosphorothioate-modified primers used in the inverse PCR are listed in Table 1. The deletion of targeted coding regions for extracellular domains 2–3 or 4–5 was confirmed by DNA sequencing.

### CMV infection assays

The SGHPL-4, HTR-8/SVneo, and MRC-5 cells seeded in 8-well chamber culture slides (BD Biosciences) were grown to 80–90% confluence, and then inoculated with cell-free CMV (Merlin wild type, Merlin ∆UL128, Merlin ∆UL130, Merlin ∆UL131A, TB40E wild type, AD169, or Towne) at a multiplicity of infection of 1 pfu/cell. Cells were incubated at 37 °C with 5% CO_2_ for 3 h, then washed once with 1 x PBS to remove the virus inoculum and incubated in appropriate growth medium (F-10 Ham’s, RPMI-1640 or Minimum Essential Medium, with 2% (v/v) FBS). For antibody inhibition assays, cells were pre-incubated with 20 μg/ml goat anti-PDGFRα antibody (AF-307-NA, R&D systems) or normal goat serum (Sigma-Aldrich) at 37 °C for 1 h, and then inoculated with different strains of CMV (Merlin wild type, Merlin ∆UL128, Merlin ∆UL130, Merlin ∆UL131A, TB40E wild type, AD169, or Towne). After 3 h inoculation, cells were washed once with PBS and incubated in fresh media containing the same concentration of PDGFRα antibody or goat serum control. For ligand competition assays, cells were pre-incubated with 20 ng/ml PDGF-AA ligand (R&D systems) at 4 °C for 1 h, and then inoculated with CMV strains at 37 °C with 5% CO_2_ for 3 h. After initial inoculation, cells were washed once with PBS and incubated in media containing the same concentration of PDGF-AA ligand. At 2 dpi, cells were fixed and stained for CMV immediate-early IE1p72 and early pUL44 (IE/E) antigens using immunofluorescence.

### Transient transfection assays

HTR-8/SVneo trophoblasts grown in 8-well chamber culture slides (BD Biosciences) were transfected with 0.32 μg of various PDGFRα expression constructs (PDGFRα WT, PDGFRα truncated, ∆D2&3, or ∆D4&5) using Lipofectamine 2000 (Life Technologies). At day 2 post-transfection, cells were infected with Merlin ∆UL128 mutant, and then analysed for PDGFRα and CMV IE/E expressions at 2 dpi. For western blotting, HTR-8/SVneo trophoblasts grown in 6-well culture plates were transfected with 4 μg of PDGFRα expression constructs, and cells were harvested at day 2 post-transfection for protein extraction and analysis.

### Indirect immunofluorescence analysis

Immunofluorescence staining was performed as previously described^69^. CMV infection and/or PDGFRα expression was detected using mouse anti-HCMV immediate early (IE1p72) and early (pUL44) antibody cocktail (IE/E; clones DDG9 and CCH2; Dako)/goat anti-PDGFRα antibody (AF-307-NA, R&D systems) and Alexa Fluor 594 donkey anti-mouse/Alexa Fluor 488 donkey anti-goat secondary antibody (Life Technologies). Nuclei were counterstained with ProLong Gold Antifade Reagent containing DAPI (Life Technologies). Immunofluorescence images were taken at room temperature (21 °C) using a Nikon DS camera unit attached to a Nikon Eclipse E400 microscope with Y-FL Epi Fluorescence emission unit. Infectivity of CMV variants were calculated as percentage of IE/E-positive nuclei in randomly selected fields under 200 × magnification (n = 15 from three separate experiments).

### Membrane protein extraction, SDS-PAGE and western blotting

The SGHPL-4, HTR-8/SVneo, MRC-5 and RPE-1 cells seeded in 25 cm^2^ culture flasks were grown to approximately 90% confluence. Membrane protein extraction was performed using the “ProteoExtract native Membrane Protein Extraction Kit (M-PEK, Calbiochem, UK)”, with slight modifications to manufacturer’s protocol. Briefly, the cell monolayer was washed twice with 2 ml ice cold M-PEK wash buffer (Calbiochem), and incubated in 2 ml of M-PEK extraction buffer I containing protease inhibitor for 10 min at 4 °C under gentle agitation. Following 10 min incubation, the supernatant containing soluble proteins was discarded. The cell monolayer was then removed gently using a cell scraper, and cells were further incubated in 300 µl of M-PEK extraction buffer II containing protease inhibitor for 30 min at 4 °C, with gentle agitation on rotary mixer. Insoluble material was removed by centrifugation at 16,000 g, 4 °C for 15 min. The supernatant containing membrane fraction enriched in integral membrane and membrane-associated proteins was collected, and boiled in 2 × laemmli buffer for 10 min.

Western blotting was performed as previously described^69^ using goat anti-PDGFRα (R&D systems) or rabbit anti-caveolin-1 (Abcam) primary antibodies and HRP-conjugated anti-goat (R&D systems) or donkey anti-rabbit (Thermo Scientific) secondary antibodies. Protein bands were visualised by chemiluminescence using Image Lab 4.1 software (Bio-Rad) and densitometry of immunostaining was performed using ImageJ software.

### Statistical analysis

The statistical significance of differences in infectivity was determined using the two-tailed Mann-Whitney U test analysis, with a P-value of less than 0.001 considered highly significant, and a P-value of less than 0.05 considered statistically significant. Analysis was performed using SPSS version 19.0 (SPSS Inc, Chicago).

## Results

### CMV gH/gL/pUL128-pUL131A complex is essential for infection of villous HTR-8/SVneo, but non-essential for infection of extravillous SGHPL-4 trophoblasts

**Figure 1:**
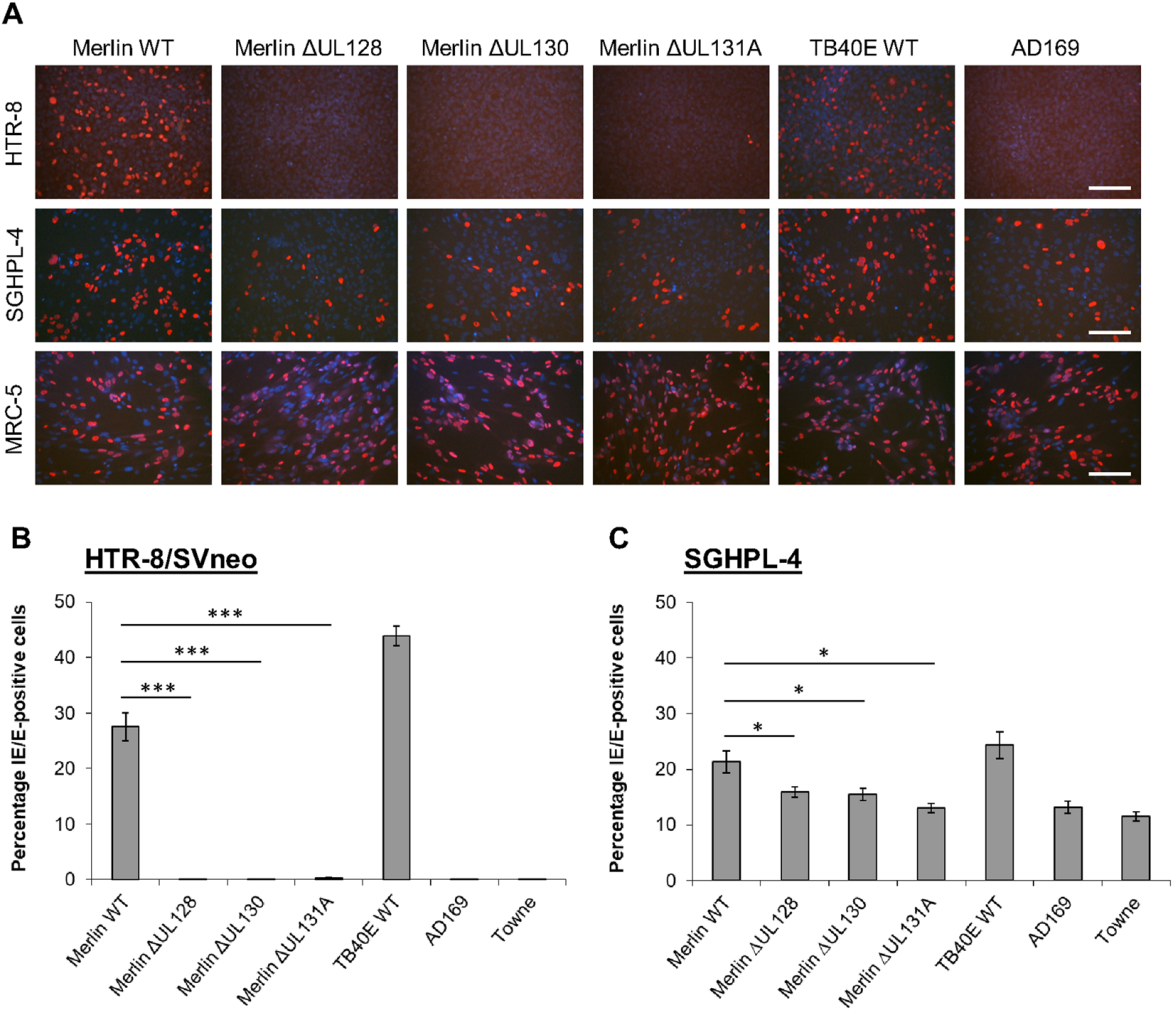
Infection efficiencies of various CMV strains in extravillous SGHPL-4 and villous HTR-8/SVneo trophoblasts. (**A)** HTR-8/SVneo trophoblasts, SGHPL-4 trophoblasts, and MRC-5 fibroblasts (control) infected with indicated CMV strains at moi of 1 pfu/cell. Viral IE/E antigens (red); Cell nuclei (blue). Scale bars represent 100 μm. Infection efficiencies of CMV strains in HTR-8/SVneo **(B)** and SGHPL-4 **(C)** cells are presented as percentage of viral IE/E-positive nuclei in randomly selected fields (*n* = 15 from three independent experiments). Mean values and standard error of the mean are shown. *P < 0.05; ***P < 0.001.

Inoculation of MRC-5 fibroblasts with CMV strains Merlin, Merlin ∆UL128, Merlin ∆UL130, Merlin ∆UL131A, TB40E wild type, AD169, and Towne (serving as a control for infectivity) resulted in similar levels of infection between the strains. Approximately, 70% of fibroblasts expressed CMV immediate-early/early (IE/E) antigens at 2 days post-infection (dpi) (Fig. 1A). Only the wild-type strains of Merlin and TB40E were able to infect HTR-8/SVneo trophoblast cells (Fig. 1A,B). CMV strains unable to assemble the gH/gL/pUL128-pUL131A complex (Merlin ∆UL128, Merlin ∆UL130, Merlin ∆UL131A, AD169, Towne), did not infect HTR-8/SVneo cells. In contrast, wild-type Merlin and TB40E, as well as CMV mutants lacking the pentameric complex were able to infect SGHPL-4 trophoblast cells (Fig. 1A,C). However, infection with wild-type Merlin in SGHPL-4 trophoblasts was significantly more efficient than the Merlin UL128-UL131A-deletion mutant counterparts (21.3% versus 15.8% IE/E-positive cells, P = 0.042). These data indicate that the gH/gL/pUL128-pUL131A complex is essential for infection of villous HTR-8/SVneo trophoblasts, and the pentameric complex contributes to efficient infection of extravillous SGHPL-4 trophoblasts, although it is not essential for infection of these cells.

### PDGFRα receptor is differentially expressed in extravillous SGHPL-4 and villous HTR-8/SVneo trophoblasts

**Figure 2:**
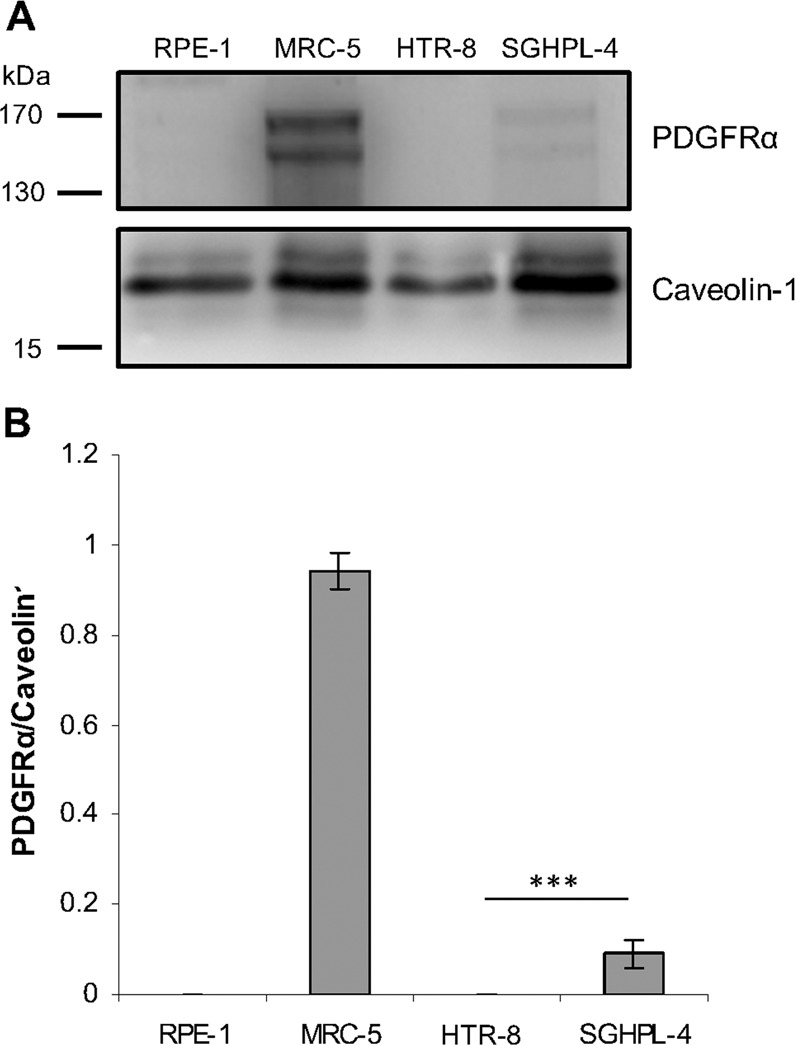
Expression levels of PDGFRα receptor in HTR-8/SVneo and SGHPL-4 trophoblasts. (**A)** Membrane lysates of RPE-1 (control), MRC-5 (control), HTR-8/SVneo, and SGHPL-4 cells were subject to Western blot analysis using PDGFRα and caveolin-1 antibodies. Full-length blots are presented in Supplementary Fig. S1. **(B)** Densitometric analyses of PDGFRα expression levels relative to caveolin-1 in different cell lines. PDGFRα/Caveolin-1 values from three independent experiments are presented as mean ± standard error of the mean. ***P < 0.001.

Since different trophoblast subpopulations may differentially express cell surface receptors, we next analysed the expression of PDGFRα, a receptor for entry into fibroblast and glioma cells. Western blot analysis of membrane fractions demonstrated high level expression of PDGFRα in MRC-5 fibroblasts, whilst extravillous SGHPL-4 cells exhibited low level PDGFRα expression on their membranes (Fig. 2A). In contrast, PDGFRα was not detected in the membrane lysates of RPE-1 or villous HTR-8/SVneo cells (Fig. 2A). Densitometric analyses were performed next to semi-quantify the expression levels of PDGFRα relative to caveolin-1 in the different cell lines. PDGFRα/caveolin-1 expression ratio was significantly higher in SGHPL-4 cells, compared with HTR-8/SVneo trophoblasts (P < 0.001) (Fig. 2B).

### PDGFRα contributes to the entry of CMV into extravillous SGHPL-4 trophoblasts in strains lacking the pentameric complex more than strains with intact pentameric complexes

**Figure 3:**
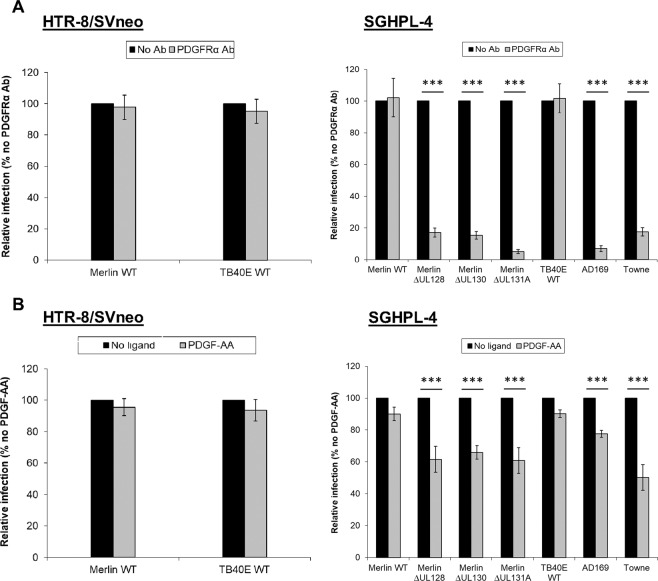
Effects of PDGFRα neutralising antibody and PDGF-AA ligand on infection of HTR-8/SVneo and SGHPL-4 trophoblasts. HTR-8/SVneo and SGHPL-4 trophoblasts treated with PDGFRα-specific antibody **(A)** or PDGF-AA ligand **(B)** were infected with indicated CMV strains, and then CMV IE/E antigens were detected using immunofluorescence. Relative infection is calculated by comparing the IE/E-positive cells in antibody-/ligand-treated samples versus no treatment controls (results from three separate experiments). Mean values and standard error of the mean are shown. ***P < 0.001.

In order to determine whether PDGFRα is involved in CMV infection of trophoblast cells, CMV entry assays were performed in the presence of PDGFRα-specific antibody or recombinant PDGF-AA ligand. Blocking with PDGFRα neutralising antibody did not reduce entry of wild-type CMV strains Merlin and TB40E into SGHPL-4 or HTR-8/SVneo trophoblasts (Fig. 3A). However, PDGFRα neutralisation with specific antibody in SGHPL-4 cells blocked the entry of CMV strains lacking gH/gL/pUL128-pUL131A complex, including Merlin ∆UL128, Merlin ∆UL130, Merlin ∆UL131A, AD169, and Towne (Fig. 3A). SGHPL-4 trophoblasts were also treated with PDGFRβ-specific antibody (served as antibody control) to determine the specificity of neutralisation effects observed with PDGFRα antibody. Neutralisation with PDGFRβ antibody did not block the entry of wild-type Merlin or Merlin ∆UL128 strains, whilst the treatment with PDGFRα specifically blocked the entry of Merlin ∆UL128 into SGHPL-4 trophoblast cells (data not shown). In addition, SGHPL-4 and HTR-8/SVneo cells were treated with 20 ng/ml dose of PDGF-AA ligand, and infection efficiencies of CMV strains were analysed. There was no evidence for inhibition of wild type Merlin and TB40E entry into SGHPL-4 or HTR-8/SVneo trophoblasts upon treatment with PDGF-AA (Fig. 3B). Similar to the PDGFRα neutralisation experiments, treatment with PDGF-AA reduced the entry of gH/gL/pUL128-pUL131A-deficient CMV strains into SGHPL-4 trophoblasts (Fig. 3B). However, the levels of inhibition on entry of CMV mutants were less pronounced with PDGF-AA ligand competition compared to receptor neutralisation using PDGFRα-specific antibody.

### Transient expression of PDGFRα promotes the entry of CMV UL128-UL131A mutants into villous HTR-8/SVneo trophoblasts

**Figure 4:**
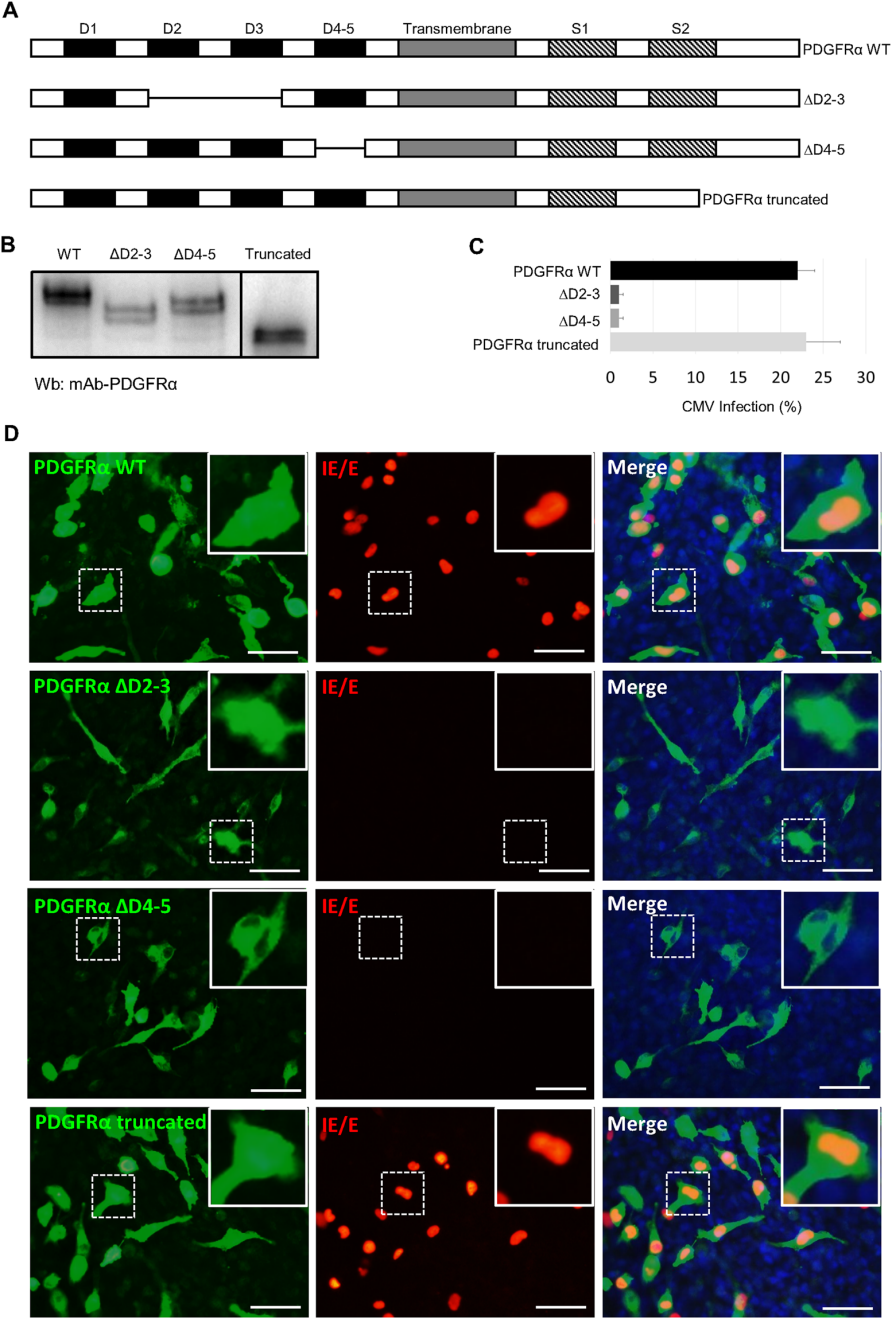
Transient expression of PDGFRα receptors in HTR-8/SVneo trophoblasts. (**A)** Schematic diagram of PDGFRα cDNA clones, representing wild type (PDGFRα WT), C-terminal truncated version (PDGFRα truncated), and extracellular domain deletion mutants (ΔD2-3, ΔD4-5). Extracellular domains (D1-5), transmembrane region, and tyrosine kinase segments (S1-2) encoded by the cDNA clones are illustrated in the diagram. **(B)** Western blot analysis of membrane fractions from HTR-8/SVneo trophoblasts transiently expressing PDGFRα receptors (PDGFRα WT, ΔD2-3, ΔD4-5 and PDGFRα truncated). Full-length blots are presented in Supplementary Fig. S2. **(C)** HTR-8/SVneo trophoblasts transiently expressing PDGFRα were infected with Merlin ∆UL128 mutant, and analysed for cellular PDGFRα and viral IE/E expressions using indirect immunofluorescence. Results show percentage of infected cells as mean ± standard deviation (*n* = 3). **(D)** Representative images of immunofluorescence staining with inset images illustrating enlargement of the representative areas. Scale bars represent 100 μm.

The effect of PDGFRα on viral entry into placental trophoblasts was also investigated using transient expression of PDGFRα in villous HTR-8/SVneo trophoblasts, which are known to be deficient for the receptor. HTR-8/SVneo trophoblasts were transfected with a wild-type PDGFRα expression plasmid (PDGFRα WT)^70^ or with a commercially obtained PDGFRα expression plasmid lacking the second tyrosine kinase segment (PDGFRα truncated; Fig. 4A). This truncated form of PDGFRα retains important autophosphorylation sites at tyrosine residues Y572, Y574, Y720, Y742, and Y754, whilst lacking Y988 and Y1018. HTR-8/SVneo trophoblasts were also transfected with wild-type PDGFRα expression plasmid where coding regions for extracellular domains 2–3 or 4–5 were deleted (PDGFRα mutants, ΔD2-3 and ΔD4-5).

Western blot analysis using polyclonal antibody raised against extracellular domains 1–5 of PDGFRα, detected PDGFRα WT (~170 kDa), PDGFRα truncated (~130 kDa), PDGFRα ΔD2-3 (~155 kDa), and PDGFRα ΔD4-5 (~160 kDa) proteins in the membrane lysates of HTR-8/SVneo cells transiently expressing respective receptors (Fig. 4B). This is consistent with predicted molecular weight of these PDGFRα receptor proteins, and also showed that the deletion of D2-3 or D4-5 did not affect cell surface expression of these mutated PDGFRα receptors. In addition to western blot analysis, immunofluorescence staining was also performed to examine transient expression of different PDGFRα receptors in HTR-8/SVneo trophoblasts. A semi-quantitative comparison of PDGFRα expression by immunofluorescence showed more efficient expression of PDGFRα WT (27 ± 2%) and PDGFRα truncated (25 ± 3%), compared with PDGFRα ΔD2-3 (17 ± 2%) and PDGFRα ΔD4-5 (15 ± 1%) mutant receptors (mean ± SD).

Transient expression of PDGFRα WT or PDGFRα truncated in HTR-8/SVneo cells promoted the entry of CMV mutants lacking gH/gL/pUL128-pUL131A complex (Merlin ∆UL128, Merlin ∆UL130, Merlin ∆UL131A) (Fig. 4C,D), consistent with the carboxy-terminal half of the receptor not playing an important role in entry of CMV mutants lacking the pentameric complex. There was no significant increase in entry of wild type CMV strains containing functional pentameric complex, upon transient expression of PDGFRα WT or PDGFRα truncated receptors (data not shown). In contrast, transient expression of PDGFRα mutants (ΔD2-3 or ΔD4-5) did not promote the entry of CMV mutants lacking gH/gL/pUL128-pUL131A complex (Fig. 4C,D). The lower transfection efficiencies of ΔD2-3 and ΔD4-5 constructs are unlikely to affect the determination of infection efficiencies due to the almost complete lack of infection in these transfected cells (0.1 ± 0.05% of cells infected). These results indicate extracellular domains 2-5 of PDGFRα are required for infection of placental trophoblasts with CMV strains unable to assemble the pentameric complex.

## Discussion

The ability of CMV to infect diverse cell types of normal placenta, and move across from mother to fetus, is an important factor in transplacental transmission of this DNA virus, and may be important for other vertically transmitted viruses. The mechanism by which CMV infects placental trophoblasts is less well understood than understanding of CMV entry pathways into epithelial, endothelial and fibroblast cells. Using two trophoblast cell lines, extravillous SGHPL-4 cells and villous HTR-8/SVneo cells, we demonstrated that CMV entry into trophoblast cells involves the viral pentameric complex (gH/gL/pUL128-pUL131A) and cellular PDGFRα.

Broad cellular tropism of clinical CMV strains *in vivo* is facilitated by the presence of various receptor binding proteins mediating entry into different cell types. One essential virion component expressed in clinical CMV strains, is the pentameric complex of gH/gL/pUL128-pUL131A. Wild type CMV strains including Merlin, TB40E, and TR have been shown to express substantial amounts and differential ratios of gH/gL/pUL128-pUL131A pentameric complexes in the virion envelope^28,38,71^. This pentameric protein complex is necessary for entry into epithelial cells, endothelial cells, monocytes, and dendritic cells^27–29,31,72,73^. Laboratory strains of CMV such as AD169 and Towne cannot assemble gH/gL/pUL128-pUL131A due to mutations within the UL128-UL131A genes^23,29^. These laboratory-adapted CMV strains have lost the ability to infect epithelial cells, endothelial cells, monocytes, and dendritic cells *in vitro*, but maintain the ability to infect diploid fibroblasts.

The present study demonstrated the gH/gL/pUL128-pUL131A complex is essential for infection of villous HTR-8/SVneo trophoblasts, whilst it is non-essential for infection of extravillous SGHPL-4 trophoblasts since CMV strains lacking the pentameric complexes were able to infect SGHPL-4 cells (Fig. 1). These results suggest different trophoblast cell types may differentially express cellular receptors specific for CMV infection in a pentameric complex (gH/gL/pUL128-pUL131A)-dependent and -independent manner. This hypothesis is compatible with inconsistencies of infection efficiencies observed in previous studies on infection of primary and other trophoblasts with AD169 and Towne strains^16–21^. Discrepant infectivities were also noted in studies comparing the efficiency of trophoblast cell infection by clinical and laboratory strains. In one study, no differences in infectivity were observed between three clinical CMV isolates, and laboratory strains AD169 and Towne^17^, whilst another showed a significantly more efficient infection of villous and cell column cytotrophoblasts with VR1814 clinical strain (pentameric complex intact wild type) compared to AD169^74^. Notably, using different CMV strains (VR1814, AD169, TB40E, TB40E ∆UL131A), and neutralising antibody against viral envelope gB or pUL130/pUL131A, Zydek and colleagues^43^ demonstrated that CMV gB, but not the viral pentameric complex is necessary for infection of placental trophoblast progenitor cells, which give rise to various trophoblast cell types.

The cellular growth factor receptor, EGFR was reported as a CMV receptor through its interaction with gB^45^ although subsequent studies have demonstrated that EGFR is not required for CMV entry into fibroblasts, epithelial cells or endothelial cells^58,75^. In other reports, integrins α2β1, α6β1, and αVβ3 were shown to promote CMV entry into cells through binding with disintegrin-like domain of gB^49,55^. Blocking with peptide containing gB disintegrin-like domain or antibodies raised against this peptide inhibited CMV infection in human fibroblasts, indicating the importance of gB in entry into these cells^55^. Accordingly, Maidji and colleagues^57^ investigated the expression of potential CMV receptors in various populations of cytotrophoblasts and reported distinct viral replication sites in the placenta that correlate with co-expression of EGFR and integrins α1β1, αVβ3, and αVβ6. Although the mechanistic roles of EGFR and certain integrin receptors in CMV entry is unclear, it is likely that co-expression of these receptors promote the engagement of virions to trophoblast cells, thereby triggering gB to fuse with plasma membrane for virus entry. In another study, Wille and colleagues demonstrated that gH/gL complex binds to cellular receptor(s) to trigger gB, and gB acts as the viral fusion protein rather than a receptor-binding protein to promote CMV entry into cells^42^.

Soroceanu *et al*. were the first to implicate PDGFRa as a receptor for entry of clinical and laboratory CMV strains into human fibroblasts and glioma cells^46^. However, a study by Vanarsdall and colleagues^58^ showed that PDGFRα promotes entry of CMV mutants lacking the pentameric complex into epithelial and endothelial cells, whilst it is not necessary for entry of CMV TR strain, a virus which is able to assemble the pentameric complex. Recent studies have shown a direct interaction between the gO subunit of gH/gL/gO and PDGFRα^51^ and, in conflict with Soroceanu *et al*.^46^., that PDGFRα activation is not necessary for CMV infection^59^. The significance of PDGFRα is particularly interesting since only a subpopulation (10–15%) of term placental cytotrophoblasts have been reported to express PDGFRα on the cell surface^62^. This variability in expression of PDGFRα is reflected in our study using continuous cell lines derived from different trophoblast cell types, with SGHPL-4 extravillous trophoblasts able to express PDGFRα at low levels, whilst HTR-8/SVneo villous trophoblasts do not express detectable levels of PDGFRα (Fig. 2A). CMV entry assays involving the blockage of PDGFRα in SGHPL-4 trophoblasts suggest PDGFRα is an important receptor for entry of CMV strains unable to assemble the pentameric complex (Fig. 3). The fact that PDGFRα antibody or PDGF-AA did not affect the entry of wild-type CMV strains suggests the pentameric complex provides an alternate pathway into trophoblast cells during the blockage of functional PDGFRα. Consistent with our study, Vanarsdall and colleagues^58^ demonstrated that PDGFRα antibodies or PDGF-AA ligand did not block wild-type CMV TR entry into fibroblasts, epithelial and endothelial cells.

Transient expression of PDGFRα (carboxy-terminal truncated version) in epithelial and endothelial cells has been shown to enhance the entry of CMV strains lacking the pentameric complex, as well as the entry of wild type CMV TR strain^58^. In this study, transient expression of PDGFRα wild type or carboxy-terminal truncated version of the receptor in HTR-8/SVneo trophoblasts equally permitted the entry of CMV strains lacking the pentameric complex (Fig. 4). These results indicate that the second tyrosine kinase segment of the PDGFRα receptor is not involved in entry of CMV strains unable to assemble the pentameric complex. However, there was no further increase in entry of CMV strains containing functional pentameric complex upon over-expression of PDGFRα in HTR-8/SVneo trophoblasts (data not shown). These results may indicate that CMV preferably utilises the pentameric complex for entry into villous HTR-8/SVneo trophoblasts. Alternatively, this could be due to Merlin virions containing significantly more gH/gL/pUL128-pUL131A than other envelope glycoprotein complexes^38^, thereby limiting CMV entry into trophoblast cells via different pathways. Interestingly, transient expression of mutant PDGFRα receptors (lacking extracellular domains 2–3 or domains 4–5) in HTR-8/SVneo trophoblasts did not permit the entry of pentameric-deficient CMV strains, indicating extracellular domains 2–5 of PDGFRα are involved in CMV entry into trophoblasts. These results are supported by recent findings demonstrating the extracellular domain 3 of PDGFRα contribute to wild type Merlin virus entry in PDGFRα-KO fibroblast cells^60^.

It has been demonstrated that ARPE-19 epithelial cells become resistant to infection with CMV TR strain upon transient expression of the pentameric gH/gL/pUL128-pUL131A complex^39^. This suggests the pentameric protein complex binds to cell-specific receptors that are required for entry into ARPE-19 cells, and therefore producing interference. However, co-expression of PDGFRα and gH/gL/pUL128-pUL131A complexes resulted in efficient entry of CMV TR into ARPE-19 cells, indicating expression of PDGFRα overcomes the interference produced by the pentameric complexes^58^. This evidence in combination with our observations that transient expression of PDGFRα in HTR-8/SVneo trophoblasts allowed the entry of CMV strains lacking the pentameric complex, suggests PDGFRα is involved in CMV entry pathway that is independent of gH/gL/pUL128-pUL131A-mediated entry. Using chemical inhibitors targeting various stages of endocytic entry, Vanarsdall and colleagues^58^ showed PDGFRα promotes the entry of CMV TR into epithelial cells via dynamin-dependent endocytosis of the virion, followed by pH-independent fusion with endosomal membranes, although this pathway is yet to be confirmed in trophoblast cells. On the other hand, Soroceanu and colleagues^46^ showed that PDGFRα directly interacts with gB for viral attachment/entry into host cells, suggesting a potent neutralising antibody against gB may be sufficient to block CMV entry via PDGFRα-mediated pathway.

The viral pentameric complex components, gH and pUL130, have been reported to induce high titre antibodies, with antiserum to these proteins have been demonstrated to effectively neutralise CMV entry into epithelial cells^44,76^, and are proposed as important targets for CMV vaccine. As PDGFRα provides an additional CMV entry pathway into trophoblasts, specific inhibitor(s) of this pathway, in combination with potent neutralising antibodies against viral pentameric complex and gB may provide a more effective prevention of CMV infection in placental trophoblast cells, and possibly other placental cell types. In a further study, chemically engineered sulfated glucans were also shown to have high antiviral activity against CMV at the stage of viral entry into human fibroblasts^77^, and may be promising candidates for drug development and antiviral strategies.

In summary, this study demonstrates CMV entry into placental trophoblast involves the viral pentameric complex of gH/gL/pUL128-pUL131A, and the cellular PDGFRα receptor. These findings suggest multiple therapeutic targets are required for prevention of CMV infection in diverse placental cell types, which may facilitate treatment strategies for prevention of CMV transmission during pregnancy.

## Supplementary information

Supplementary information

## Data Availability

The datasets generated during and/or analysed during the current study are available from the corresponding author on reasonable request.
