# Supplementary material for: Differential Expression of PDGF Receptor-α in Human Placental Trophoblasts Leads to Different Entry Pathways by Human Cytomegalovirus Strains
Source: Sci Rep. 2020 Jan 23;10:1082. doi: 10.1038/s41598-020-57471-3 (PMC6978357; doi:10.1038/s41598-020-57471-3)
Supplement: Supplementary file 1 — Supplementary information [file 41598_2020_57471_MOESM1_ESM.pdf]

Differential Expression of PDGF Receptor-α in Human Placental Trophoblasts Leads to Different Entry Pathways by Human Cytomegalovirus Strains

*Zin Naing[1,3], *Stuart T Hamilton[1,2] , Wendy J. van Zuylen[1,3], Gillian Scott[1,4], William D. Rawlinson[1,2,3,4]#

*Joint first authors

[1]Serology and Virology Division, Department of Microbiology, NSW Health Pathology, Prince of Wales Hospital, Sydney, New South Wales, Australia.

[2]School of Women's and Children's Health, Faculty of Medicine, University of New South Wales, Sydney, New South Wales, Australia

[3]School of Medical Sciences, Faculty of Medicine, University of New South Wales, Sydney, New South Wales, Australia.

[4]School of Biotechnology and Biomolecular Sciences, University of New South Wales, Sydney, New South Wales, Australia.

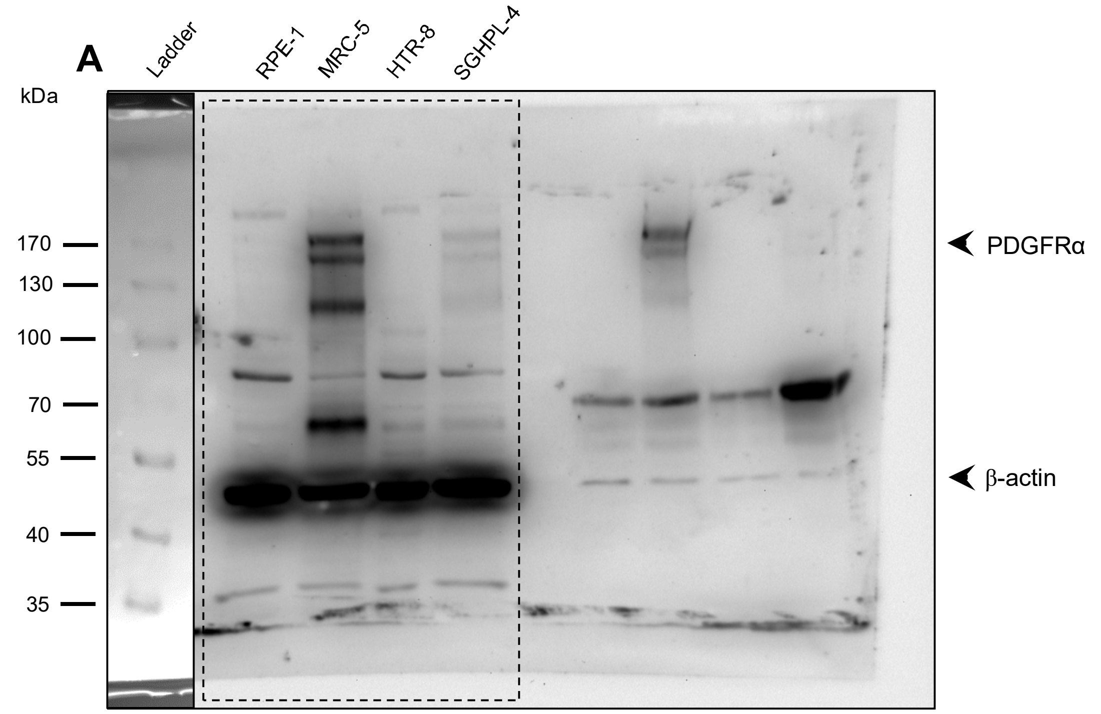

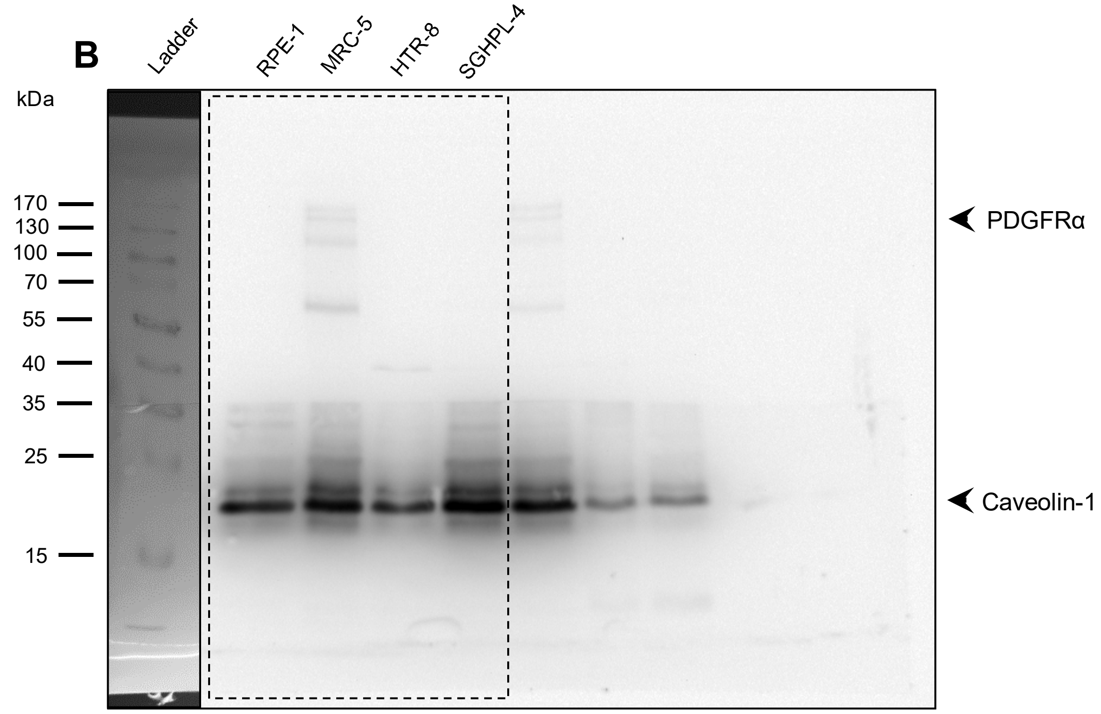

Figure S1. Full-length blots for expression levels of PDGFRα receptor in HTR-8/SVneo and SGHPL-4 trophoblasts. Membrane lysates of RPE-1 (control), MRC-5 (control), HTR-8/SVneo, and SGHPL-4 cells were subject to Western blot analysis using **(A)** PDGFRα and β-actin antibodies or **(B)** PDGFRα and caveolin-1 antibodies. Rectangle represents full-length area that is shown in the main figure.

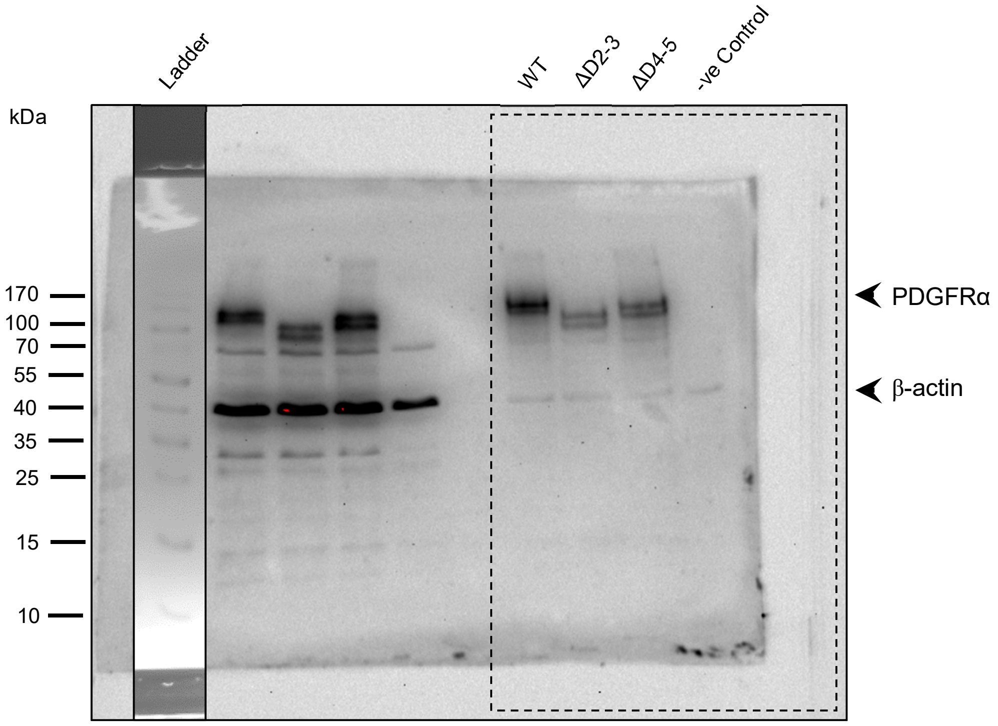

Figure S2. Full-length blot for transient expression of PDGFRα receptors in HTR-8/SVneo trophoblasts. Western blot analysis of membrane fractions from HTR-8/SVneo trophoblasts transiently expressing PDGFRα receptor constructs using PDGFRα and β-actin antibodies. Rectangle represents full-length area that is shown in the main figures.
